# Tumor mutational burden adjusted by neutrophil-to-lymphocyte ratio serves as a potential biomarker for atezolizumab-treated patients with extensive stage small cell lung cancer

**DOI:** 10.1186/s12931-024-02885-0

**Published:** 2024-06-21

**Authors:** Chenyue Zhang, Yanfei Huo, Xiaoling Shang, Tongming Zhang, Ning Tang, Haiyong Wang

**Affiliations:** 1 Department of Integrated Therapy, Fudan University Shanghai Cancer Center, Shanghai Medical College, Shanghai, China; 2 Department of Oncology, Shanghai Medical College, Fudan University, Shanghai, 200032 China; 3 Shandong Provincial Key Laboratory of Radiation Oncology, Cancer Research Center, Shandong Cancer Hospital and Institute, Shandong First Medical University, Shandong Academy of Medical Sciences, Jinan, Shandong Province 250117 China; 4 Shandong Cancer Hospital and Institute, Shandong University, Jinan, 250117 China; 5 Department of Internal Medicine-Oncology, Shandong Rizhao Port Hospital, Rizhao, 276800 China; 6 Department of Internal Medicine-Oncology, Shandong Cancer Hospital and Institute, Shandong First Medical University and Shandong Academy of Medical Sciences, Jinan, 250117 China

**Keywords:** SCLC, TMB, Neutrophil-to-lymphocyte ratio, Overall survival

## Abstract

**Background:**

There is a desperate for the identification of more accurate and efficient biomarkers for ICI responses in patients with SCLC.

**Methods:**

The data of our study was obtained from IMpower133 study. A total of 202 patients with SCLC received the treatment of placebo plus carboplatin plus etoposide (EC) while a total of 201 patients with SCLC received the treatment of atezolizumab plus EC. Overall survival (OS) was compared using Kaplan Meier analyses. Univariate and multivariate Cox regression analysis were used to determine independent prognostic variables affecting OS in patients with SCLC.

**Results:**

We have demonstrated that a higher TMB adjusted by a lower neutrophil-to-lymphocyte ratio (NLR) is significantly correlated with improved OS, in patients with SCLC subject to either atezolizumab or placebo (*P* = 0.001 for atezolizumab and *P* = 0.034 for placebo). Moreover, Cox model showed that TMB < 10 mut/Mb adjusted by NLR ≥ median was an independent factor of OS for atezolizumab-treated SCLC patients (hazard ratio [HR], 2.82; 95% confidence interval; 1.52–5.24; *P* = 0.001). Both univariate and multivariate cox regression analysis showed that for patients with SCLC harboring low NLR and high TMB, survival is significantly longer in those treated with atezolizumab than those treated with placebo. Survival benefit is significantly higher in atezolizumab-treated patients with SCLC than those treated with placebo (*P* = 0.018 for TMB cutoff = 10 mut/Mb, *P* = 0.034 for TMB cutoff = 16 mut/Mb).

**Conclusion:**

Our findings provide a promising insight into the utility of NLR-adjusted TMB in the prognosis and immune responses in patients with SCLC.

**Supplementary Information:**

The online version contains supplementary material available at 10.1186/s12931-024-02885-0.

## Introduction

Small cell lung cancer (SCLC) is a lethal disease with dismal survival. The advent of immune checkpoint inhibitors (ICI) has ushered in a new treatment paradigm and prolonged survival substantially in SCLC. Recently, the incorporation of immunotherapy to carboplatin and etoposide has resulted in boosted efficacy as compared with chemotherapy alone in SCLC [1]. The IMpower133, a trial evaluating both the efficacy and safety of atezolizumab plus carboplatin and etoposide in patients with extensive-stage SCLC (ES-SCLC), has shown that significantly longer overall survival (OS) and progression-free survival (PFS) was observed in patients with atezolizumab, thus achieving an unprecedented breakthrough in the treatment of SCLC [2]. Despite improved OS, ICI response has been found in only a small fraction of patients with SCLC. Therefore, the selection of appropriate patients with SCLC benefiting from ICI therapy is of great importance to achieve optimized clinical benefit. Several clinical characteristics such as tumor programed cell death ligand 1 (PD-L1) and tumor mutational burden (TMB) have been reported to be associated with ICI responses in SCLC [3–5]. However, these biomarkers are with limited utility and lack robust evidence for appropriate patient selection [6, 7]. Thus, there is a desperate need for exploration of more reliable biomarkers to help guide immunotherapeutic treatment in SCLC.

Neutrophil to lymphocyte ratio (NLR), an inflammatory parameter, has been adopted to predict survival and response to cancer treatments [8, 9]. Mounting evidences demonstrated an intimate association between NLR and responses to ICI among cancer patients due to its underlying role in systematic inflammation and its interaction with the immune system. A previous study demonstrated that high expression of pre-treatment NLR is linked with shorter OS and PFS and with debilitated response in patients with metastatic non-small cell lung cancer (NSCLC) undergoing nivolumab [10]. However, the predictive role of NLR in patients with SCLC undergoing ICI has been widely unknown. Moreover, the potential value of TMB after NLR adjustment in ICI response is not well elucidated either.

We first evaluated the role of TMB and NLR levels in predicting ICI among patients with SCLC. Then we analyzed the predictive value of combining TMB with NLR in patients with SCLC undergoing ICI. We showed that a higher TMB adjusted by a lower NLR is associated with prolonged survival in atezolizumab-treated patients with SCLC. Moreover, an improved survival has been found in patients with SCLC harboring higher TMB adjusted by a lower NLR among those treated with either atezolizumab or placebo. Our study has demonstrated that the NLR-adjusted TMB provides predictive utility in patients with SCLC undergoing ICI.

## Methods

### Data sources

The data of our study was obtained from IMpower133 study. The IMpower133 study is a randomized, double-blind, phase I/III study, demonstrated that adding atezolizumab to carboplatin plus etoposide (EC) for first-line treatment of ES-SCLC resulted in significant improvement in OS and PFS versus placebo plus EC. The study and data have been published, thus informed consent and ethical committee approval were not warranted. A total of 202 patients with SCLC received the treatment of placebo plus EC while a total of 201 patients with SCLC received the treatment of atezolizumab plus EC. Data collected included age, sex, race, tobacco history (TOBHX), years of smoking, Eastern Cooperative Oncology Group (ECOG), baseline sum of the longest diameters (BASLD), metastasis number, brain metastasis, liver metastasis, tumor cells/ immune cells (TC/IC), TMB and NLR. The flowchart of patients was shown in Supplementary Fig. 1.

### Patient classification

TMB is defined as the number of somatic, coding, base substitutions, and short insertion and deletions per megabase of genome examined [11]. Based on prior studies, the TMB cutoff was set at ≥ 10 mut/Mb and ≥ 16 mut/Mb indicating a positive biomarker status. NLR was determined by dividing the absolute count of neutrophils by the absolute count of lymphocytes [12] and calculated from the most recent complete blood count before treatment. The NLR cutoff was set at ≥ 3.44. In the analysis of the combined effect of NLR and TMB on OS, we assigned patients into four categories of high/low NLR and high/low TMB, using the selected cutoff of TMB = 10 mut/Mb and NLR cutoff = 3.44.

### Statistical analysis

OS was compared using Kaplan Meier analyses. Univariate cox regression analysis was used to determine independent prognostic variables affecting OS in patients with SCLC. Multivariate Cox regression analysis was conducted to analyze the hazard ratio (HR) of OS in patients with SCLC according to different clinical variables. The subgroup analysis results are presented in corresponding forest plots. All statistical analyses were performed using R (version 4.1), and a p-value < 0.05 was considered statistically significant.

## Results

### The influence of clinical characteristics on survival in patients with SCLC treated with atezolizumab plus EC

**Fig. 1:**
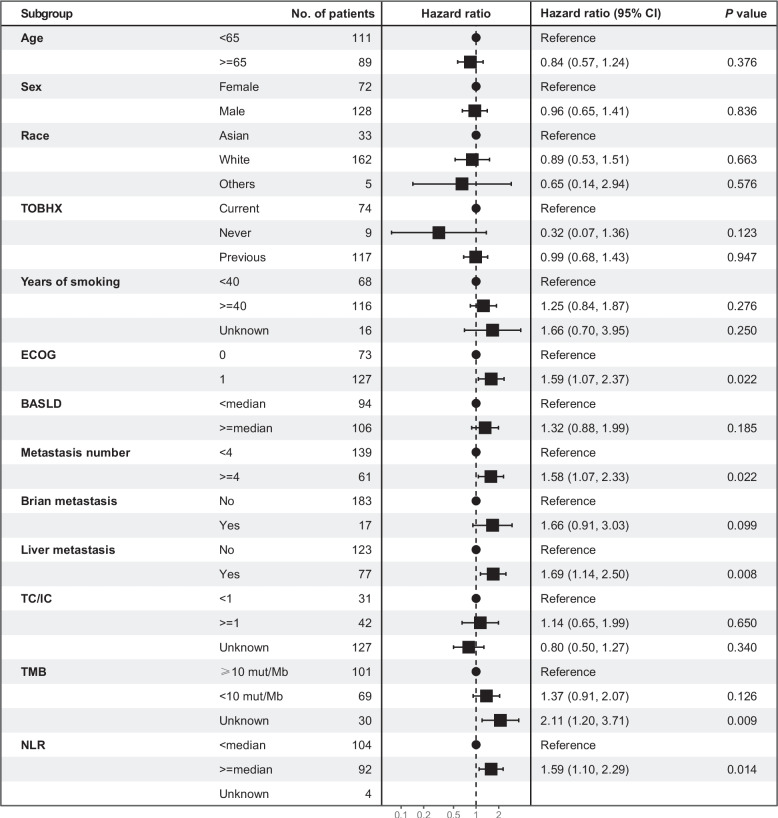
Forest plot graphs reporting the hazard ratios (HRs) for the risk of death in patients with ES-SCLC according to clinical variables. These clinical variables include age, sex, race, TOBHX, years of smoking, ECOG, BASLD, metastasis number, brain metastasis, liver metastasis, TC/IC, TMB and NLR. HR, hazard ratio

Using a Cox proportional hazards regression model, we investigated the influence of several clinical characteristics on OS: age, sex, race, TOBHX, years of smoking, ECOG, BASLD, metastasis number, brain metastasis, liver metastasis, TC/IC, TMB, NLR. In the multivariate analysis, ECOG (HR, 1.59; 95% CI, 1.07–2.37; *P* = 0.022), metastasis number (HR, 1.58; 95% CI, 1.07–2.33; *P* = 0.022), liver metastasis (HR, 1.69; 95% CI, 1.14–2.50;* P* = 0.008) and a higher NLR (HR, 1.59; 95% CI, 1.10–2.29; *P* = 0.014) were associated with worse OS, while TMB and the other clinical characteristics were not significantly associated with OS (*P* > 0.5), as demonstrated in Fig. 1.

### The role of the combination of TMB and NLR in predicting survival for patients with SCLC treated with either atezolizumab or placebo

**Fig. 2:**
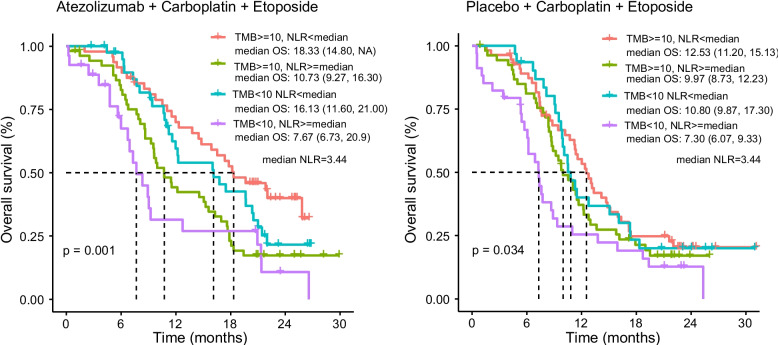
The role of the combination of TMB and NLR in predicting survival for patients with SCLC treated with either atezolizumab or standard chemotherapy. Kaplan–Meier survival estimates for OS according to the different combinations of TMB and NLR. (A) Atezolizumab cohort: TMB ≥ 10 mut/Mb and NLR < median versus TMB ≥ 10 mut/Mb and NLR ≥ median versus TMB < 10 mut/Mb and NLR < median versus TMB < 10 mut/Mb and NLR ≥ median (B) Placebo cohort: TMB ≥ 10 mut/Mb and NLR < median versus TMB ≥ 10 mut/Mb and NLR ≥ median versus TMB < 10 mut/Mb and NLR < median versus TMB < 10 mut/Mb and NLR ≥ median

Using cutoffs of TMB ≥ 10 mut/Mb, and NLR of < median for positive biomarkers, we investigated the combination of these two biomarkers in the prediction of survival among patients with SCLC. For patients with SCLC treated with atezolizumab plus EC, OS was significantly longer in patients with TMB ≥ 10 mut/Mb and NLR of < median, as compared with other groups of patients. Those harboring TMB < 10 mut/Mb and NLR ≥ median had lower OS (median OS: 7.76 months), which stands in sharp significant difference with other groups (*P* = 0.001). Additionally, 20 SCLC patients from Shandong Cancer Hospital and Institute, who received immunotherapy combined with chemotherapy, were performed to validate this result. The result showed that the OS was significantly longer in patients with TMB ≥ 10 mut/Mb and NLR of < median, as compared to those with TMB < 10 mut/Mb and NLR of ≥ median (*P* = 0.04; median OS: 17.10 months vs. NA). Due to the small sample size, there was no statistically significant difference in OS betweenpatients with TMB ≥ 10 mut/Mb and NLR of < median and the other two groups (*P* > 0.05) (Supplementary Fig. 2). Likewise, for patients with SCLC treated with EC, those with relatively higher TMB and lower NLR also improve survival than those harboring lower TMB and higher NLR (median OS: 12.53 moths vs. 7.30 months; *P* = 0.034), as shown in Fig. 2.

**Fig. 3:**
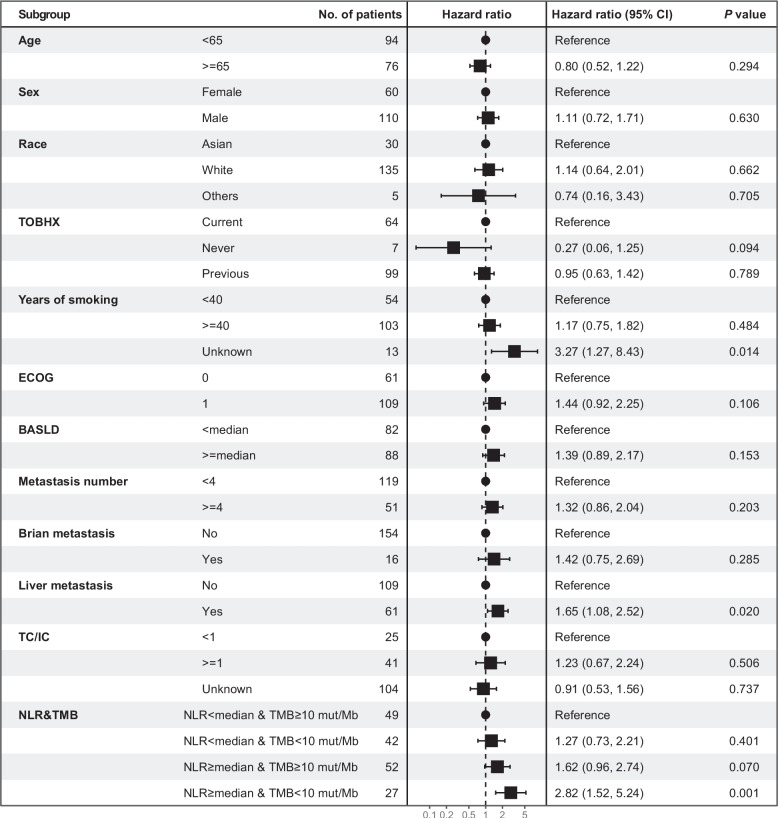
Multivariate Cox hazard regression forest plot among atezolizumab-treated patients with ES-SCLC according to clinical variables. Clinical variables include age, sex, race, TOBHX, years of smoking, ECOG, BASLD, metastasis number, brain metastasis, liver metastasis, TC/IC, NLR and TMB

**Fig. 4:**
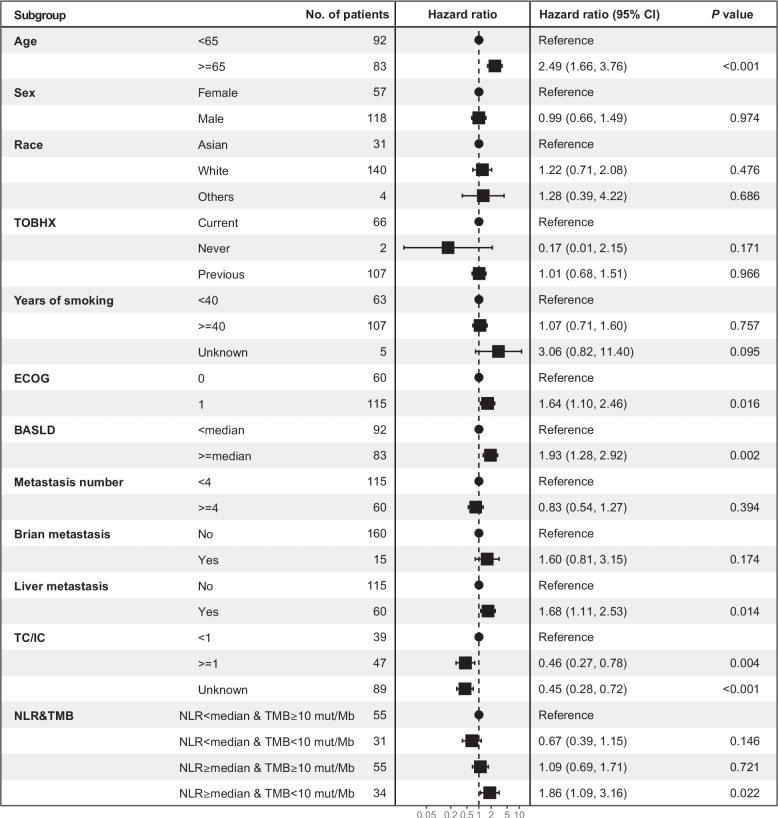
Multivariate Cox hazard regression forest plot among placebo-treated patients with ES-SCLC according to clinical variables. Clinical variables include age, sex, race, TOBHX, years of smoking, ECOG, BASLD, metastasis number, brain metastasis, liver metastasis, TC/IC, NLR and TMB

To further validate the prognostic role of the biomarker of NLR-adjusted TMB, multivariate Cox hazard regression model was adopted in patients with SCLC undergoing atezolizumab. Results have shown that the NLR-adjusted TMB maintained its significant impact on survival. For atezolizumab-treated patients with SCLC, those harboring NLR ≥ median and TMB < 10 mut/Mb have significantly worse OS as in comparison with those with NLR < median and TMB ≥ 10 mut/Mb (HR, 2.82; 95% CI, 1.52–5.24; *P* = 0.001) (Fig. 3). Likewise, for patients with SCLC undergoing placebo, those harboring NLR ≥ median and TMB < 10 mut/Mb have numerically worse OS as in comparison with those with NLR < median and TMB ≥ 10 mut/Mb (HR, 1.86; 95% CI, 1.09–3.16; *P* = 0.022) (Fig. 4). In conclusion, these results have demonstrated that the biomarker of NLR-adjusted TMB has prognostic role in patients with SCLC undergoing atezolizumab.

### Survival difference between patients with SCLC treated with either atezolizumab or placebo

**Fig. 5:**
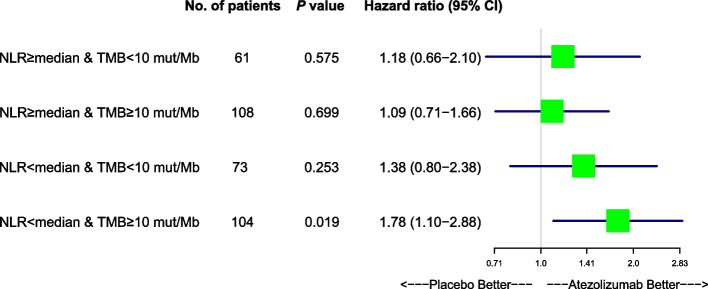
Univariate Cox hazard regression forest plot for patients with SCLC harboring low NLR and high TMB high NLR and low TMB versus high NLR and high TMB versus low NLR and low TMB versus low NLR and high TMB. Hazard ratio, 95% confidence interval and p values are shown

**Fig. 6:**
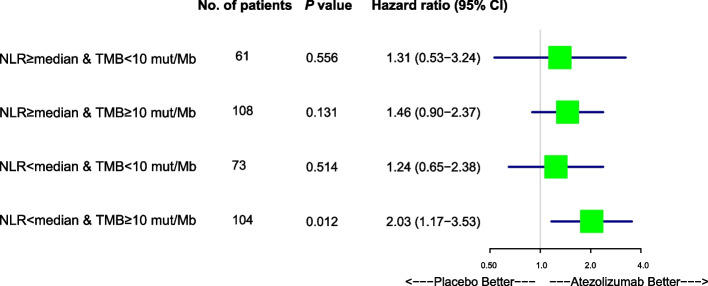
Multivariate Cox hazard regression forest plot for patients with SCLC harboring low NLR and high TMB high NLR and low TMB versus high NLR and high TMB versus low NLR and low TMB versus low NLR and high TMB. Hazard ratio, 95% confidence interval and p values are shown

Since we have proven that patients with SCLC harboring high TMB adjusted by low NLR have longer survival than any other groups both in the atezolizumab-treated and placebo-treated patients with SCLC. Next, we would like to compare the survival between atezolizumab-treated and placebo-treated patients with SCLC harboring high TMB and low NLR. Univariate cox regression analysis showed that for patients with SCLC harboring low NLR and high TMB (N = 104), survival is significantly longer in those treated with atezolizumab than those treated with placebo (HR, 1.78; 95% CI, 1.10- 2.88; *P* = 0.019) (Fig. 5). Next, multivariate cox regression analysis showed that for patients with SCLC with low NLR and high TMB (N = 104), survival is significantly longer in those treated with atezolizumab than those treated with placebo (HR, 2.03; 95% CI, 1.17- 3.53; *P* = 0.012) (Fig. 6). Using cutoffs of TMB = 10 mut/Mb, the combination of high TMB with low NLR showed significantly increased OS in patients treated with atezolizumab compared with those treated with placebo, as shown in Kaplan Meier analysis (*P* = 0.018). Similarly, using cutoffs of TMB = 16 mut/Mb, a high TMB adjusted by a low NLR also significantly improve OS in atezolizumab-treated patients with SCLC as in comparison with those treated with placebo (*P* = 0.043) (Supplementary Fig. 3). In conclusion, these results demonstrated that high TMB adjusted by low NLR indicates longer survival in patients with SCLC treated with atezolizumab than those treated with placebo.

## Discussion

The identification of appropriate patients with SCLC benefiting from ICI has remained to be solved. A cohort of biomarkers have been reported to predict prognosis in clinical practice, which includes PD-L1, TMB, tumor infiltrating lymphocytes (TILS), T-cell receptor clonality, and gene signatures [13–16]. However, despite some observed correlations, there is a lack of concrete support for the use of PD-L1 expression levels in SCLC as a predictive biomarker for atezolizumab response [3]. Moreover, exploratory subgroup analyses performed in the IMpower 133 study showed that TMB levels at a cutoff of 10 or 16 mutations per megabase had no clear predictive power for atezolizumab response. Other biomarkers have not been fully identified in predicting the survival of anti-PD-L1 antibody atezolizumab plus EC among patients with SCLC. With betterment of these biomarkers and the exploration of others, screening of reliable and accurate biomarkers is pivotal for patients with SCLC undergoing ICI. The present study examined the prognostic utility of TMB after NLR adjustment in patients with SCLC treated with the current standard front-line therapy, atezolizumab with carboplatin and etoposide. These results demonstrated that a higher TMB adjusted by a lower NLR could predict survival for ES-SCLC patients treated with atezolizumab.

IMpower133 has demonstrated a significantly longer OS with the addition of atezolizumab to standard chemotherapy in the first-line treatment of ES-SCLC as compared with mere chemotherapy. Median OS was significantly extended to 12.3 months in patients receiving atezolizumab with carboplatin and etoposide, as significantly longer than 10.3 months in those receiving carboplatin and etoposide [17].

In the present study, we have analyzed data from IMpower 133 via multiple Cox regression model among patients with SCLC who received atezolizumab plus standard chemotherapy. A relatively higher NLR was associated with worsened OS, as consistent with previous studies suggesting the association between elevated NLR and worse outcomes among patients treated with ICI. Considering their respective association of TMB and NLR with OS in patients with SCLC treated with atezolizumab plus carboplatin and etoposide, we next analyzed survival by stratifying patients with SCLC according to different values of TMB and NLR. Not surprisingly, we showed that for patients undergoing atezolizumab plus chemotherapy, TMB ^high^ NLR ^low^ patients achieve more survival benefit than patients with other forms of combinations of TMB and NLR. Meanwhile, a statistically significant longer OS was observed in TMB ^high^ NLR ^low^ patients with SCLC treated with standard chemotherapy. In conclusion, the prognostic value of NLR-adjusted TMB as a biomarker in patients with SCLC has been revealed, regardless of whether they received atezolizumab or placebo.

There have been several studies reporting the role of NLR in the prediction of ICI-treated lung cancer patients. In a study led by Diem, an increase in NLR is associated with debilitated response rates in patients with metastatic NSCLC treated with nivolumab [10]. Similarly, in another study led by Li, it is revealed that pretreatment NLR is associated with the outcomes among ICI-treated advanced NSCLC patients [18]. Only until recently, there have been several studies reporting the utility of NLR in patients with SCLC undergoing ICI. It has been found that a decreased level of NLR among early-stage SCLC patients in response to anti-PD-1/PD-L1 [19]. Another study showed that patients with SCLC harboring low NLR may benefit most from ICI treatment [20]. The mechanism underlying the association between a low NLR and longer survival outcomes remains unclear. NLR, defined as neutrophil to lymphocyte ratio, has been recognized as a reliable biomarker to evaluate the inflammatory status of immune system since both neutrophils and lymphocytes are major components responsible for the host defense [21, 22]. With the ability to protect the host from invasive pathogens, neutrophils are abundantly concentrated in the tumor, which participates in tumor immune escape [23]. Furthermore, tumor-associated neutrophiles have been demonstrated to promote tumor progression. And immature neutrophils serve as another subtype of neutrophils and were considered to function as myeloid derived suppressor cells (MDSCs) in tumors, which incurs an immunosuppressive milieu in the tumor microenvironment [24, 25]. And lymphocytes play a central role in effective antitumor immunity due to their potent capability to kill tumor cells [26, 27]. Patients with an increased level of NLR demonstrated exhausted T cell immunity, highlight the importance of NLR in ICI treatment [28].

The association of high TMB with enhanced efficacy of ICI in patients with SCLC might be explained by the fact that a high TMB can generate neoantigens, thus boosting immunogenicity. However, it has to be noted that, as revealed in the subgroup analyses conducted in the IMpower 133 study, TMB at a cutoff of either 10 or 16 mut/Mb had no predictive value for response to atezolizumab. It is assumed that this may lie in the fact of highly active and myelosuppressive nature of platinum and etoposide, which may influence the predictive power of TMB greatly.

Moreover, the comparison of survival between atezolizumab plus carboplatin and carboplatin provided us deeper insights. Interestingly, we found that patients with a higher TMB adjusted by a lower NLR achieve better survival from atezolizumab than placebo alone. These findings first proposed that the NLR-adjusted TMB can be employed to differentiate patients with SCLC obtaining more clinical benefit from atezolizumab than mere placebo. In clinical practice, clinicians could select those patients who benefit from immunotherapy, based on both NLR and TMB levels. Those ES-SCLC patients with a higher TMB adjusted by a lower NLR would benefit more from immunotherapy compared to chemotherapy.

Our present study has several strengths. First, the data from our study was extracted from IMpower 133 trial and was preliminarily validated using external data. Second, we not only focus on SCLC patients undergoing atezolizumab plus platinum doublet treatment, but also on those with standard chemotherapy. We have found a trend towards better OS among atezolizumab-treated patients than chemotherapy-treated patients using TMB after NLR adjustment, which may influence our clinical decision making for therapeutic treatments for patients with SCLC. Third, we employed a cutoff of TMB at both 10 mut/Mb and 16 mut/Mb, which elevated the robustness of our study.

Undeniably, this study has some unignored limitations. We do not analyze the correlation between TMB and NLR in the present study. Second, the median value for NLR in the included population was set as the cut-off, which allows us to obtain a sound result in our study. However, it should be confirmed further that whether this cutoff is widely recognized or adopted for SCLC. Third, a comparison analysis in survival between mere TMB, NLR and their integration can lend more credibility to our study. Additionally, Although external data was used for preliminary validation, due to the small sample size, there was no statistically significant difference in OS betweenpatients with TMB ≥ 10 mut/Mb and NLR of < median and the other two groups. Further prospective studies would be necessary to generate sufficient samples to validate these conclusions.

Despite the aforementioned limitations, we are the first to illustrate the prognostic capability of NLR-adjusted TMB in response to atezolizumab-treated SCLC from a randomized clinical trial population. The NLR-adjusted TMB was proven to be predictive for SCLC patients treated with either atezolizumab or placebo. In particular, patients with a higher TMB after a lower NLR adjustment derived a greater benefit from atezolizumab as compared with placebo. In addition, the NLR-adjusted TMB could be adopted for patient selection for different survival in response to atezolizumab. Meanwhile, the different OS stratification indicates a possible predictive role for NLR-adjusted TMB among patients with SCLC. Nevertheless, further validations are also warranted to ensure the clinical utility and precision of NLR-adjusted TMB in larger prospective SCLC cohorts.

### Supplementary Information

Supplementary Material 1: Supplementary Figure 1. The flowchart of patients.

Supplementary Material 2: Supplementary Figure 2.Validation of TMB and NLR combined prediction of survival in SCLC patients undergoing ICI treatment from Shandong Cancer Hospital and Institute.

Supplementary Material 3: Supplementary Figure 3. Comparison of survival between atezolizumab-treated and chemotherapy-treated patients with SCLC harboring high TMB adjusted by low NLR (A) Kaplan Meier curves for survival in atezolizumab and placebo group with TMB= 10 mut/Mb as the cutoff. (B) Kaplan Meier curves for survival in atezolizumab and placebo group with TMB= 16 mut/Mb as the cutoff.

## Data Availability

No datasets were generated or analysed during the current study.
