# Supplementary figures and images for: Tumor mutational burden adjusted by neutrophil-to-lymphocyte ratio serves as a potential biomarker for atezolizumab-treated patients with extensive stage small cell lung cancer
Source: Respir Res. 2024 Jun 21;25:253. doi: 10.1186/s12931-024-02885-0 (PMC11191253; doi:10.1186/s12931-024-02885-0)

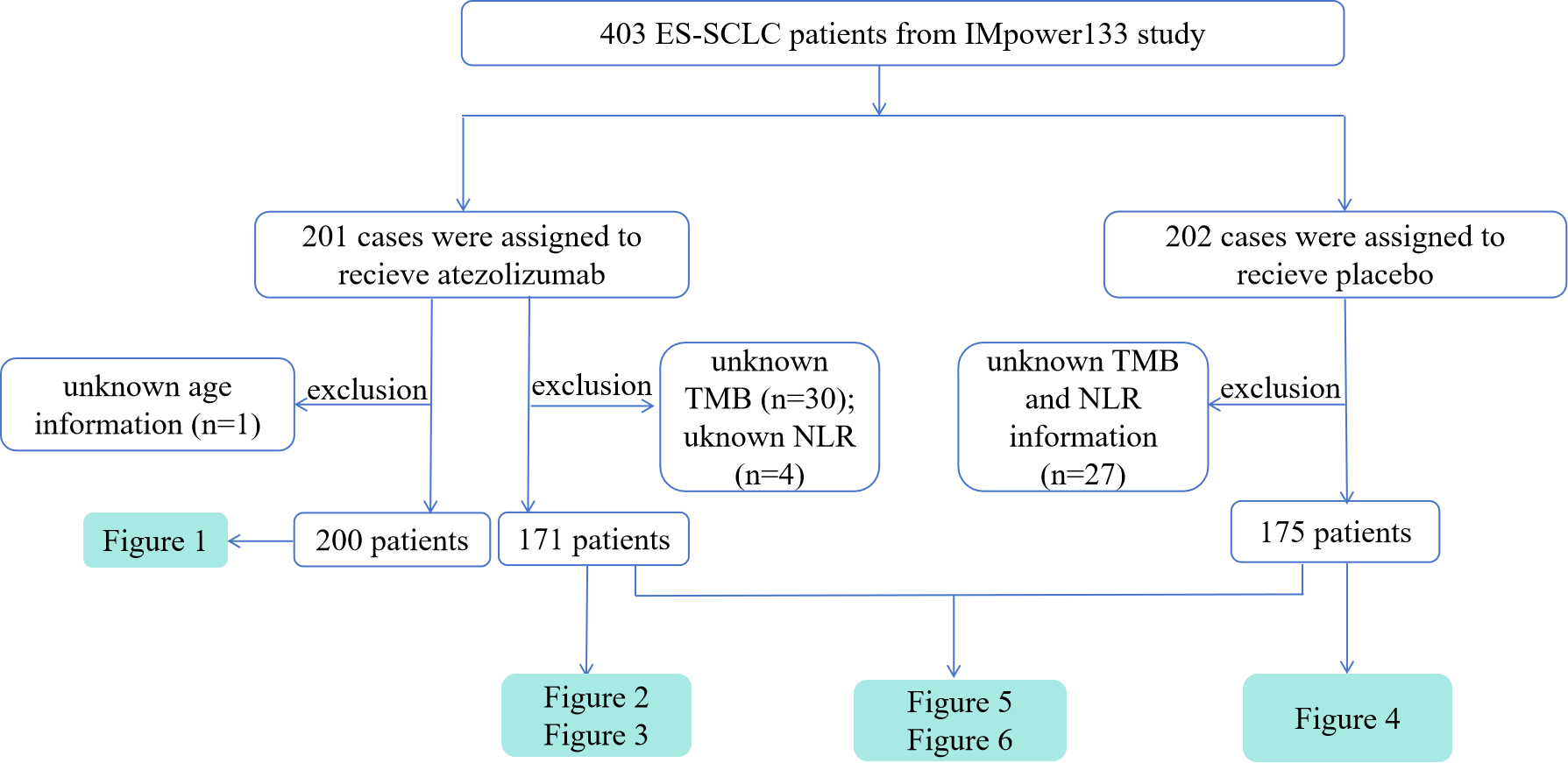

Supplement: Supplementary file 1 — Supplementary Material 1: Supplementary Figure 1. The flowchart of patients. [file 12931_2024_2885_MOESM1_ESM.png]

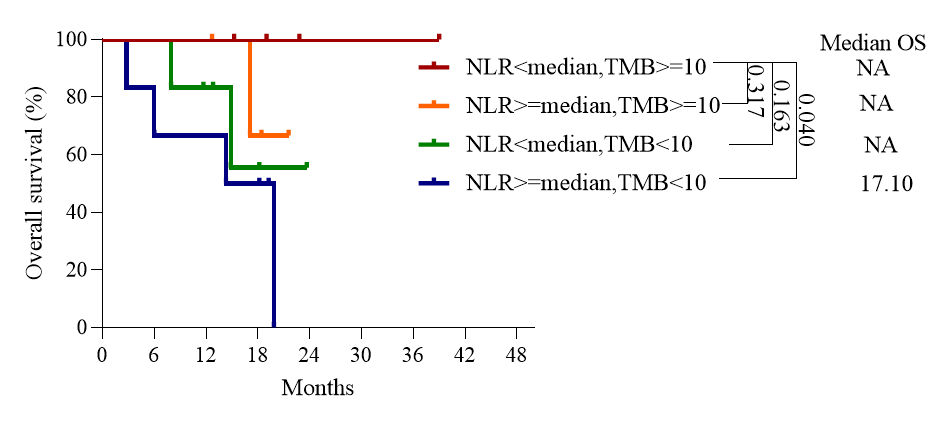

Supplement: Supplementary file 2 — Supplementary Material 2: Supplementary Figure 2.Validation of TMB and NLR combined prediction of survival in SCLC patients undergoing ICI treatment from Shandong Cancer Hospital and Institute. [file 12931_2024_2885_MOESM2_ESM.png]

NLR < median & TMB  $\geq 10$  (median NLR=3.44)

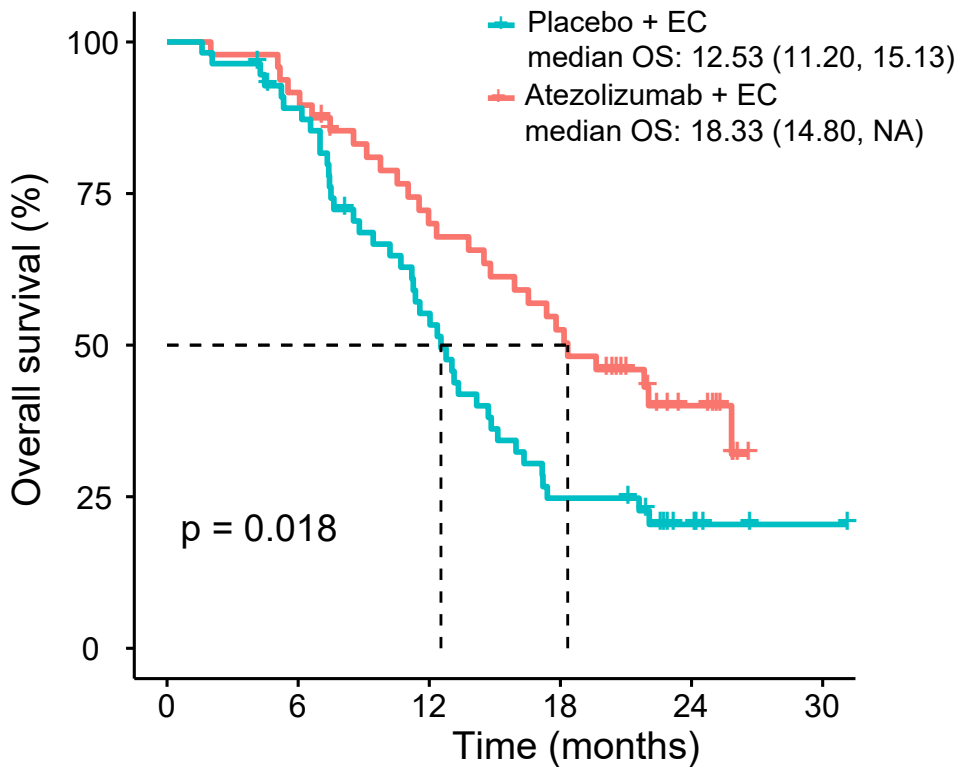

NLR < median & TMB  $\geq 16$  (median NLR=3.44)

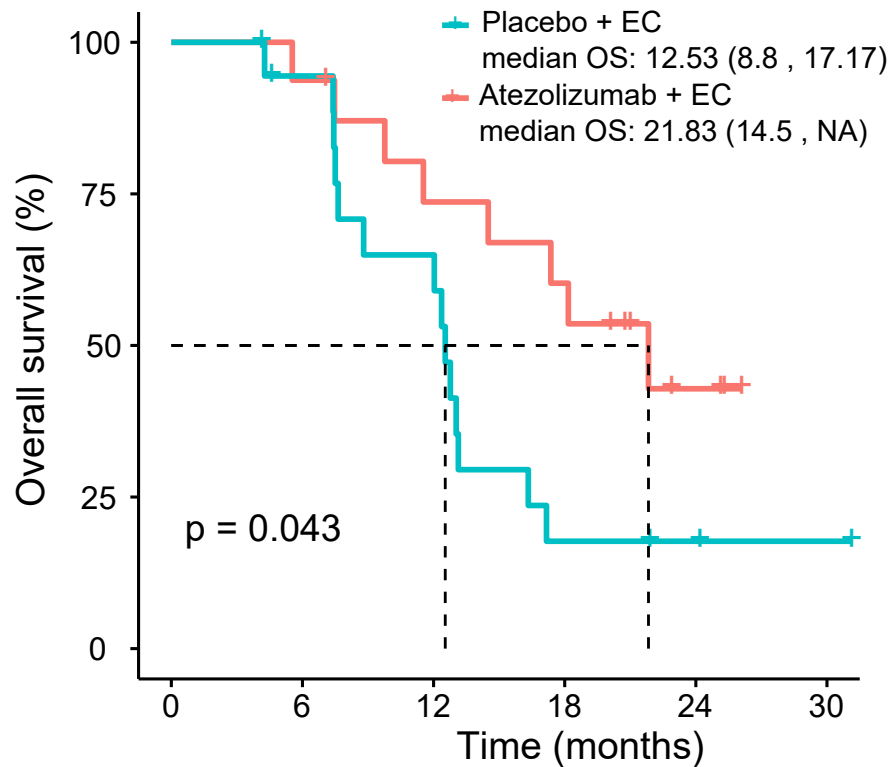

Supplement: Supplementary file 3 — Supplementary Material 3: Supplementary Figure 3. Comparison of survival between atezolizumab-treated and chemotherapy-treated patients with SCLC harboring high TMB adjusted by low NLR (A) Kaplan Meier curves for survival in atezolizumab and placebo group with TMB= 10 mut/Mb as the cutoff. (B) Kaplan Meier curves for survival in atezolizumab and placebo group with TMB= 16 mut/Mb as the cutoff. [file 12931_2024_2885_MOESM3_ESM.pdf]
